# Incidence, predictors and vascular sequelae of distal limb ischemia in minimally invasive cardiac surgery with femoral artery cannulation: an observational cohort study

**DOI:** 10.1007/s00380-023-02241-0

**Published:** 2023-02-01

**Authors:** Angelique Ceulemans, Ruben Derwael, Jeroen Vandenbrande, Katelijne Buyck, Ine Gruyters, Michiel Van Tornout, John M. Murkin, Pascal Starinieri, Alaaddin Yilmaz, Björn Stessel

**Affiliations:** 1 Department of Anesthesiology and Critical Care, Jessa Hospital, Stadsomvaart 11, 3500 Hasselt, Belgium; 2 Department of Anesthesiology and Perioperative Medicine, University Hospitals-LHSC, University of Western Ontario, London, ON Canada; 3 Department of Perfusion, Jessa Hospital, Hasselt, Belgium; 4 Department of Cardiac Surgery, Jessa Hospital, Hasselt, Belgium

**Keywords:** Distal limb ischemia, Minimally invasive cardiac surgery, Femoral artery cannulation near-infrared spectroscopy, Cardiopulmonary bypass

## Abstract

Literature regarding monitoring and consequences of distal limb ischemia due to femoral artery cannulation for Minimally Invasive Cardiac Surgery (MICS) remains limited. The primary objective was to determine its incidence, defined as a ≥ 15% difference in regional Oxygen Saturation (rSO_2_) lasting ≥ four consecutive minutes between the cannulated and non-cannulated limb. The secondary objectives included: determination of distal limb ischemia, defined as a Tissue Oxygenation Index (TOI) < 50% in the cannulated limb, identification of predictors for distal limb ischemia, determination of a possible association of NIRS-diagnosed ischemia with acute kidney injury, and the need for vascular surgery up to six months after cardiac surgery. A prospective, observational cohort study with blinded rSO_2_-measurements to prevent intraoperative clinical decision-making. A single-center, community-hospital, clinical study. All consecutive patients ≥ 18 years old, and scheduled for predefined MICS. Patients underwent MICS with bilateral calf muscle rSO_2_-measurements conducted by Near-Infrared Spectroscopy (NIRS). In total 75/280 patients (26.79%) experienced distal limb ischemia according to the primary objective, while 18/280 patients (6.42%) experienced distal limb ischemia according to the secondary objective. Multivariate logistic regression showed younger age to be an independent predictor for distal limb ischemia (*p* = 0.003). None of the patients who suffered intraoperative ischemia required vascular surgery within the follow-up period. The incidence of NIRS-diagnosed ischemia varied from 6.4% to 26.8% depending on the used criteria. Short and long-term vascular sequelae, however, are limited and not intraoperative ischemia related. The added value of intraoperative distal limb NIRS monitoring for vascular reasons seems limited. Future research on femoral artery cannulation in MICS should shift focus to other outcome parameters such as acute kidney injury, postoperative pain or paresthesias.

## Introduction

Cannulation of the peripheral vessels is a commonly used technique for connecting the patient to the Cardio-Pulmonary Bypass (CPB) in Minimally Invasive Cardiac Surgery (MICS) [1]. The femoral vein and artery are the preferred peripheral cannulation sites [2]. However, peripheral cannulation raises concerns regarding both retrograde flow to the body, and the risk of compromising antegrade blood flow to the cannulated limb [3]. Distal limb ischemia, due to cannula-related obstruction, is a known entity in patients on Veno-Arterial Extracorporeal Membrane Oxygenation (VA-ECMO) [4, 5]. A recent systematic review reported an incidence of distal limb ischemia ranging from eight to 42% in this population [6]. Moreover, hazardous consequences, such as compartment syndrome and limb loss, are described in [7–9]. In contrast, little is known regarding sequelae of distal limb ischemia, due to cannula-related obstruction, after MICS [10]. Available studies in MICS patients are limited, though reported incidences vary from 4.3% to 31.5% [11–13]. Despite this notable incidence, no prospective study investigated vascular sequelae after cannulation in the prolonged postoperative phase. This is not futile since retrospective reports mention postoperative clinical complication rates to vary from 0.7% to 7% after femoral artery cannulation in MICS. Over 50% of sequelae were noticed only after hospital discharge and 66% of these patients required a second hospitalization for surgical resolution [9, 14]. Also, the relationship between intraoperative NIRS monitoring and postoperative vascular complications has never been investigated before. The primary objective of this proposed observational cohort study was to determine the incidence of distal limb ischemia, assessed with Near-Infrared Spectroscopy (NIRS), in 280 patients undergoing MICS with femoral artery cannulation. Secondary objectives were to determine the incidence of distal limb ischemia using measurements on the cannulated limb only [15], to identify predictors of distal limb ischemia, and to screen for both short and long-term vascular sequelae and postoperative kidney function. Due to the lack of hazardous complications described in MICS literature, we hypothesize a low correlation between intraoperative distal limb NIRS-diagnosed ischemia and vascular complications in the short and long term.

## Materials and methods

A prospective, observational cohort study including 280 patients undergoing MICS with femoral artery cannulation was performed at the Jessa Hospitals, Belgium. Approval from the ethical committee of the hospital was obtained on September 12, 2019 (B243201941236). The study was conducted in accordance with the declaration of Helsinki. All patients provided written informed consent. All consecutive patients ≥ 18 years old scheduled for MICS, with femoral artery cannulation, were screened for eligibility. Eligible patients were approached and screened for in- and exclusion criteria. Exclusion criteria included: a medical history of distal limb amputation, calf muscle atrophy due to neuromuscular disorder or muscle diseases, Stanford type A aortic dissection, body mass index (BMI) > 40, allergy for NIRS electrode/band aids, presentation for revision surgery < 72 h after primary surgery, and postoperative need for intra-aortic balloon pump and/or VA-ECMO. The study was initiated in November 2019. The last patient was included in January 2021. All data were collected by our department’s research team. Follow-up was carried out by telephone call, and the last patient was contacted six months after surgery, being July 27, 2021. Our study was powered for evaluation of predictors and a sample size estimation was performed based on available literature. A previous study showed that approximately 25% of patients suffered from clinically relevant ischemia in the cannulated limb during the VA-ECMO run [16]. Aiming for 10 patients with ischemia per variable, 40 patients were needed to identify a variable as a risk factor. Since seven variables were analyzed, a total of 280 patients were included in the study.

All patients were contacted six months after surgery, and were interviewed for the need for additional vascular surgery within the six-month follow-up period. Three telephonic attempts were made to contact the patient. In addition, charts of all contacted patients were reviewed to supplement anamnestic information.

### Near-Infrared spectroscopy

NIRS is a continuous, non-invasive tool for monitoring the fraction of oxygenated versus deoxygenated hemoglobin in tissue. The difference in wavelength between emitted and absorbed infrared light is measured, whereof the rSO2 is calculated [17]. Despite known disadvantages, such as spatial resolution and tissue heterogeneity, rSO_2_ measurements showed a high sensitivity for immediate detection of clamping of the femoral artery [18].

**Fig. 1:**
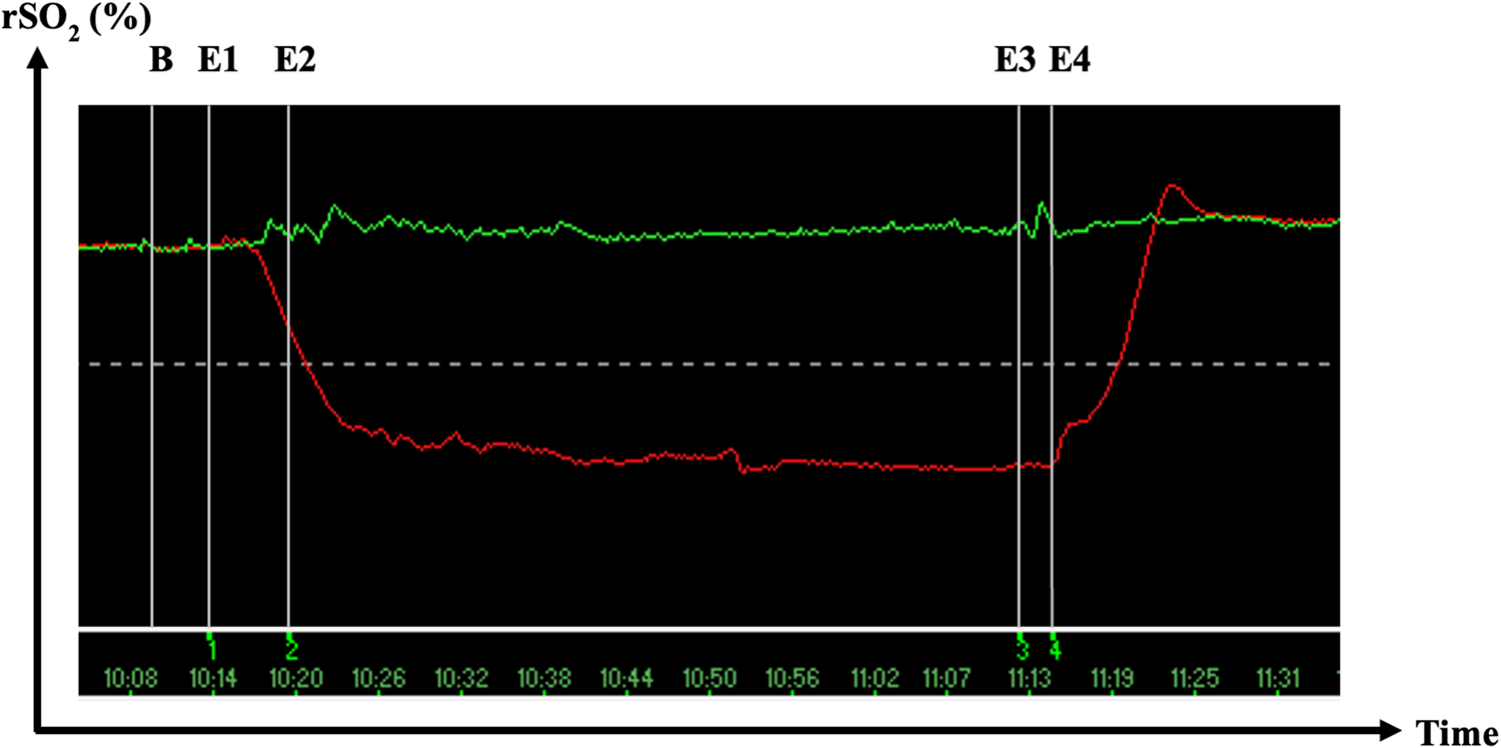
Representative image of a regional Oxygen Saturation (rSO_2_) measurement in time (minutes) with Near-Infrared Spectroscopy (NIRS). During the NIRS-measurement, five main events were captured: baseline (B) measurement 5 min before the occurrence of event 1; Event 1 (E1) = insertion of the femoral artery cannula; Event 2 (E2) = onset of the cardiopulmonary bypass; Event 3 (E3) = offset of the cardiopulmonary bypass; Event 4 (E4) = removal of the femoral artery cannula. These events are captured simultaneously for both limbs, whereby the red line corresponds to the NIRS-measurement of the cannulated limb, and the green line corresponds to the NIRS-measurement of the non-cannulated limb

On arrival at the operating room, patients were connected to the NIRO-200NX device (Hamamatsu^®^, Photonics K.K., Japan) with electrodes placed on top of the skin of the calf muscles bilaterally. Cerebral oximetry was monitored with the same device as routine care in our institution. Live results of the lower limb oximetry were blinded to the attending anesthesiologist, perfusionist and surgical team, to prevent clinical decision making. Five events were marked in the patient’s NIRS file: (1) insertion of the femoral artery cannula (E1), (2) onset of CPB (E2), (3) offset of CPB (E3), and (4) withdrawal of the femoral artery cannula (E4) (Fig. 1). The baseline NIRS value (B) was defined as the value five minutes before event one. A rSO_2_-differential of ≥ 15% lasting ≥ four consecutive minutes disadvantaging the cannulated limb was defined as ischemia as per primary outcome [16]. Since some advocate to only measure NIRS values in the cannulated limb, we calculated the Tissue Oxygenation Index (TOI) by dividing the lowest value by the baseline in the cannulated limb [19]. A TOI < 50% was defined as our secondary definition of ischemia [15].

### Cannulation technique

For connection to the CPB, the femoral artery and vein are cannulated in the groin. Arterial cannula size was dependent upon the required minimal flow for adequate cardiac indices, as calculated based on Body Surface Area (BSA), and upon surgical inspection of the vessel. The cannulation technique in our center is the cut-down Seldinger technique, in which a cut-down of the groin region is performed, and the guidewires are inserted under direct surgical vision of the vessel [20]. To facilitate surgical set-up, the left groin is the preferred cannulation site. Previous stenting of the left femoral artery was a relative contra-indication, albeit in these patients the right groin was cannulated [21, 22]. Furthermore, the femoral artery is never clamped and distal limb backflow cannulas are not placed in our institution during MICS.

### Data management

Clinical data were collected via paper Case Report Forms (CRFs) and archived in a secured room. Pseudonymized data were completed and collected in an encrypted excel file and transferred to an encrypted SPSS file. Anonymized rSO2-measurements were saved onto an encrypted USB and stored together with the paper CRFs. Access to all trial data was restricted to study personnel only.

### Statistical analysis

All data analyses were performed with IMB SPSS Statistics software version 27. Data are presented as means ± Standard Deviation (SD) or medians with Interquartile Range (IQR) based on the nature of the data. To assess differences in rSO_2_ between the cannulated and non-cannulated limb, an independent sample *T*-test or Mann–Whitney *U* test was used, based on the nature of the data. To assess differences in rSO_2_ between baseline and its corresponding four events, the Wilcoxon-signed rank test or paired samples T-test was used, based on the nature of the data. Uni- and multivariate binary logistic regression analyses of predefined variables were used to identify possible risk factors that might increase the likelihood of developing NIRS-diagnosed ischemia. Variables that showed *p* < 0.2 in univariate analysis were considered relevant, and were included in the multivariate analysis. Results are presented as odds ratios (OR) and their corresponding 95% confidence intervals (CI). The association between the presence of NIRS-diagnosed ischemia in the cannulated limb, and the need for additional peripheral vascular surgery within six months after the initial surgery was assessed with a Chi-square or Fisher’s exact analyses, based on the nature of the data *p*-values < 0.05 were considered statistically significant.

## Results

**Fig. 2:**
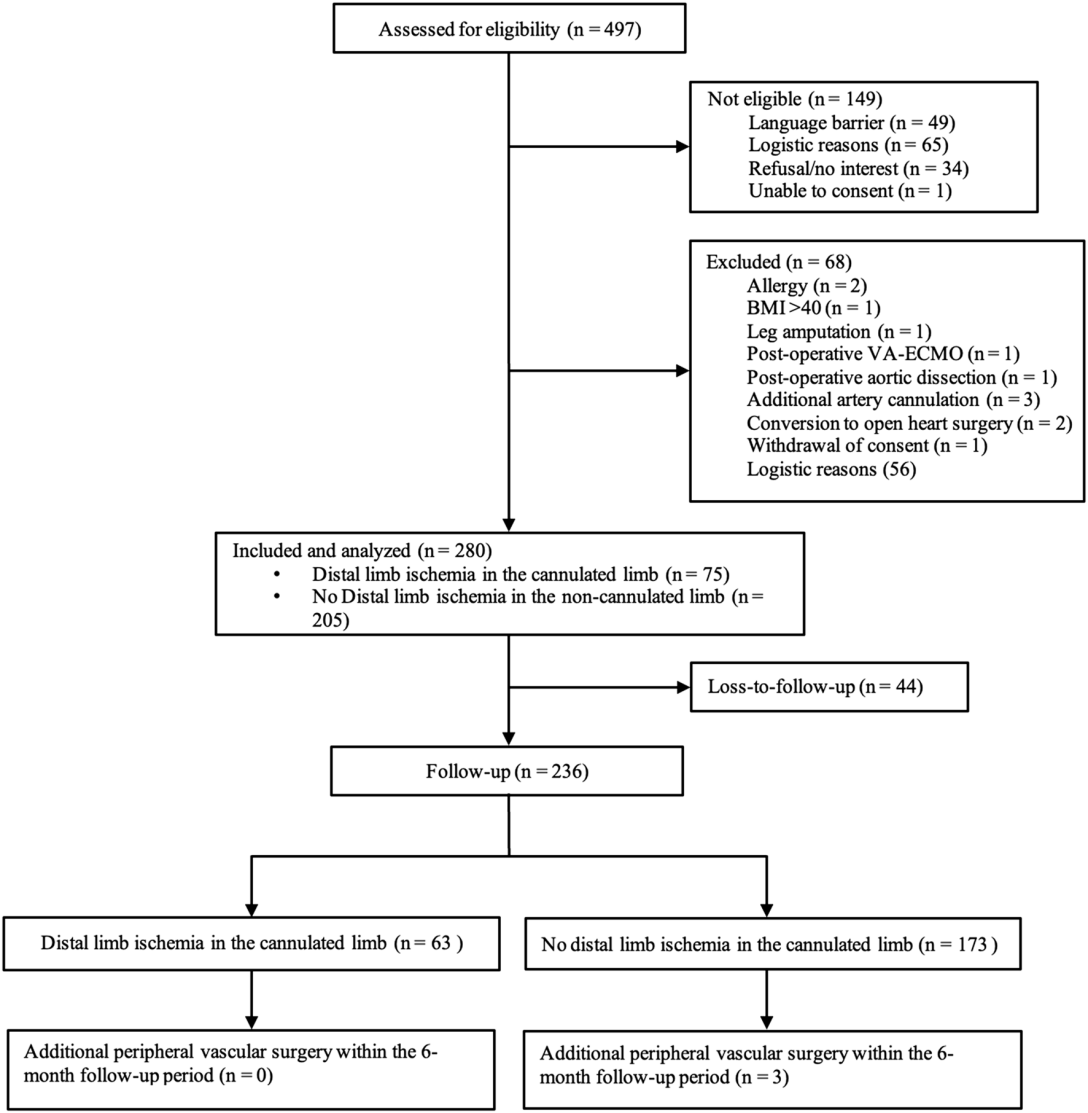
Consort flowchart. A total of 497 patients were screened for eligibility, whereof 149 were not eligible and 68 were excluded due to various reasons described above. After the removal of non-eligible and excluded patients, the estimated sample size of 280 was reached. Of all 280 patients, 44 patients could not be contacted for telephone follow-up after six months, rendering 236 patients for the follow-up statistical analysis. Of all 236 patients, 63 patients developed distal limb ischemia in the cannulated limb (defined as s a ≥ 15% difference in regional Oxygen Saturation (rSO2) lasting ≥ four consecutive minutes between the cannulated and non-cannulated limb) and 173 patients who did not develop distal limb ischemia in the cannulated limb. Among those 173 patients, three patients required additional peripheral vascular surgery within the six-month follow-up period

Between November 2019 and January 2021, a total of 497 patients scheduled for MICS with femoral artery cannulation were assessed for eligibility, of which 149 patients were not eligible, and 68 patients were excluded. In total, 280 patients were analyzed for incidence of NIRS-diagnosed ischemia and possible risk factor analysis. Six months after surgery, 236 patients could be contacted by telephone, indicating a loss-to-follow-up for long-term complications of 15.71% (Fig. 2). Of note, information on the reasons for loss-to-follow-up is largely lacking**.** The male/female ratio (%) was 75.7/24.3.

**Fig. 3:**
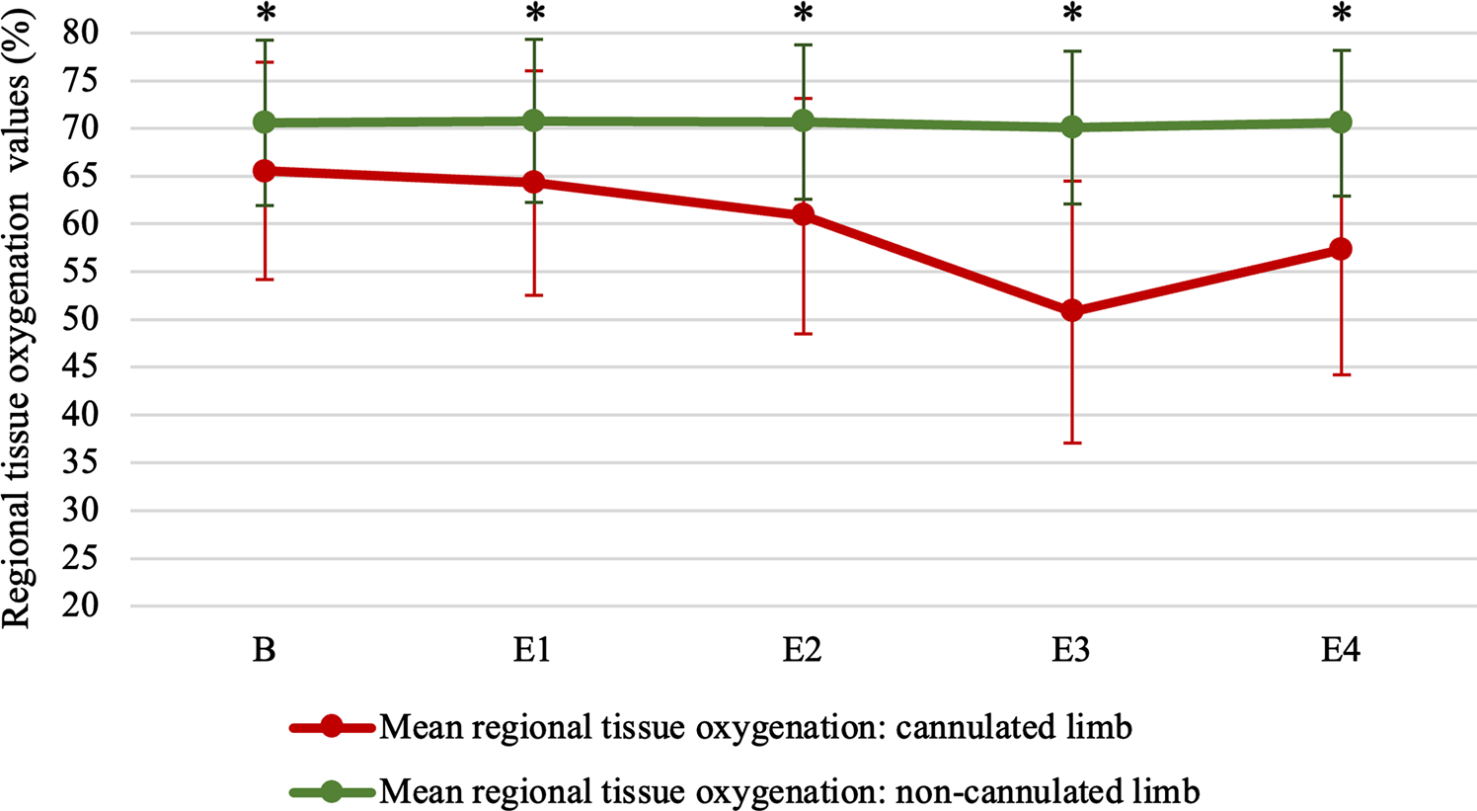
Comparison of the mean rSO2-values in all 75 patients who experienced a rSO_2_-drop ≥ 15% lasting ≥ four consecutive minutes in the cannulated limb compared to the non-cannulated limb during MICS. A parametric independent samples *T*-test was used for the comparison of baseline and all four events between the cannulated and non-cannulated limb (E1 = insertion of the femoral arterial cannula; E2 = onset of the CPB; E3 = offset of the CPB; 4 = removal of the femoral arterial cannula. Statistically significant differences are indicated with (*)

**Fig. 4:**
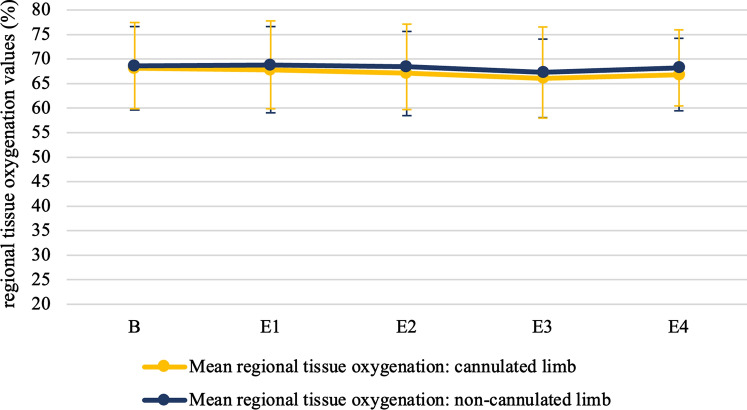
Comparison of the mean rSO_2_-values in all 205 patients who did not experienced a rSO_2_-drop ≥ 15% lasting ≥ four consecutive minutes in the cannulated limb compared to the non-cannulated limb during MICS. A parametric independent samples *T*-test was used for the comparison of baseline and all four events (E1 = insertion of the femoral arterial cannula; E2 = onset of the CPB; E3 = offset of the CPB; 4 = removal of the femoral arterial cannula. Statistically significant differences are indicated with (*)

Of the total cohort, mean patient age was 68.74 ± 10.31 years. Mean Body Surface Area (BSA) was 1.95 ± 0.21, and 7.86% had a medical history of previous peripheral vascular surgery. A total of 107 patients (38.21%) presented for minimally invasive valve surgery, 156 patients (55.71%) presented for endoscopic Coronary Artery Bypass Grafting (endo CABG), and 17 patients (6.07%) presented for combined CABG plus valve surgery. Mean duration of cannulation was 106.40 ± 42.10 min, while mean Cardiac Index (CI) on CPB was 1.91 ± 0.18 (Table 1). With the arterial cannula in place, 75 out of 280 patients had rSO_2_-differentials ≥ 15% lasting at least four consecutive minutes disadvantaging the cannulated limb. Albeit, the incidence of distal limb ischemia, as defined by our primary outcome parameter, was 26.79%. In these patients, baseline mean ± SD (%) rSO_2_-values of the cannulated and non-cannulated limb were 65.43 ± 11.38 and 70.53 ± 8.64 (*p* = 0.002), respectively. Mean ± SD rSO_2_ values at all four events of the cannulated and non-cannulated limb were: E1 = 64.04 ± 11.93 and 70.74 ± 8.49 (p < 0.01); E2 = 60.74 ± 12.31 and 70.64 ± 8.06 (*p* < 0.01); E3 = 50.34 ± 14.23 and 70.02 ± 8.00 (p < 0,001); E4 = 56.82 ± 13.65 and 70.48 ± 7.65 (*p* < 0.001), respectively (Fig. 3). The mean ± SD duration of ischemia was 73.82 ± 49.22 min. The presence of ischemia in these 75 patients was not associated with perioperative Acute Kidney Injury (AKI) (*p* = 0.433) (Table 2). The remaining 205 out of 280 patients (73.21%) who did not develop NIRS-diagnosed distal limb ischemia in the cannulated limb, based on our primary outcome parameter, showed no statistically significant difference at any event of the cannulated limb compared to the non-cannulated limb (Fig. 4). A total of 18 out of 280 patients (6.42%) had a TOI < 50% in the cannulated limb. Likewise, the presence of distal limb ischemia in these 18 patients was also not associated with perioperative AKI (*p* = 0.374) (Table 2). The predictive value of all seven variables of interest on the occurrence of NIRS-diagnosed ischemia in the cannulated limb during MICS was assessed with uni- and multivariate binary regression analyses (Table 3). The independent variables age (*p* = 0.014; OR 0.968; 95% CI 0.944–0.994), gender (*p* = 0.134; OR 0.636; 95% CI 0.352–1.149), BSA (*p* = 0.097; OR 0.331; 95% CI 0.090–1.223), and surgical type (valve surgery versus coronary artery bypass graft surgery; *p* = 0.186; OR 0.691; 95% CI 0.400–1.195) appeared to be risk factors for ischemia in the cannulated limb during MICS after univariate binary logistic regression. However, only younger age showed to be of predictive value after multivariate binary logistic regression (*p* = 0.003) (OR 0.960; CI 0.934–0.987). During the six-month follow-up period, three out of 236 patients underwent additional peripheral vascular surgery (Table 2 and 4). All were male between 61 and 73 years of age. One patient underwent resection of a lymphocele in the cannulated groin, one a percutaneous transluminal angioplasty (PTA) plus stenting for in-stent-restenosis in the non-cannulated limb. The third patient required PTA plus stenting of the common iliac artery on the cannulated side 148 days after endo-CABG. None of the three patients met NIRS-diagnosed criteria of ischemia during CPB. Zero patients from the whole cohort required fasciotomy or limb amputation in the postoperative period.

**Table 1:** Patient characteristics

	Ischemia (*n* = 75)	No Ischemia (*n* = 205)	*p*-value
Demographic characteristics			
Age (years)	66.21 ± 10.76	69.66 ± 10.00	0.013
Sex (% male)	52/75 (69.33)	160/205 (78.05)	0.132
BMI (kg/m^2^)	27.28 ± 4.61	27.67 ± 4.07	0.496
BSA (m^2^)	1.91 ± 0.20	1.96 ± 0.21	0.096
Alcohol	14/75 (18.67)	47/205 (23.04)	0.929
Smoking	16/75 (21.33)	35/205 (17.16)	0.324
Diabetes			
Type I	3/75 (4.00)	5/205 (2.44)	0.533
Type II	11/75 (14.67)	39/205 (19.02)	
Hypercholesterolemia	48/75 (64.00)	157/205 (76.59)	0.158
Arterial hypertension	44/75 (58.67)	142/205 (69.27)	0.150
Peripheral artery disease	6/75 (8.00)	24/205 (11.71)	0.513
Coronary artery disease	44/75 (58.67)	138/205 (67.32)	0.360
Chronic kidney disease	27/75 (36.00)	57/205 (27.80)	0.140
Intermittent hemodialysis	2/75 (2.67)	0/205 (0.00)	
Atrial fibrillation	9/75 (12.00)	32/205 (15.61)	0.568
Previous cardiac surgery	7/75 (9.33)	9/205 (4.39)	0.142
Femoral artery CT	0/75 (0.00)	2/205 (0.98)	1.000
Femoral artery ultrasound	0/75 (0.00)	3/205 (1.46)	0.567
NYHA-score			
I	20/75 (26.67)	66/205 (32.20)	0.774
II	42/75 (56.00)	112/205 (54.63)	
III	9/75 (12.00)	22/205 (10.73)	
IV	1/75 (1.33)	2/205 (0.98)	
Euroscore II	2.86 ± 3.26	2.85 ± 3.21	0.978
Creatinine levels (mg/dL)	1.17 ± 0.65	1.10 ± 0.47	0.359
Intraoperative characteristics			
Type of surgery			
Valve	34/75 (45.33)	73/205 (35.61)	0.298
Coronary	38/75 (50.67)	118/205 (57.56)	
Valve + coronary	3/75 (4.00)	14/205 (6.83)	
Mean cardiac index on CPB	1.90 ± 0.18	1.82 ± 0.18	0.322
Duration of cannulation (min)	108.77 ± 41.51	105.53 ± 42.38	0.569
CPB configured T°	35.01 ± 0.12	35.01 ± 0.29	0.970
Lowest Hct on CPB	30.42 ± 5.35	31.01 ± 4.98	0.386
Distal limb backflow cannula	0/75	0/205	/
Arterial cannula size (Fr)			
15	3/75 (4.00)	3/205 (1.46)	0.362
17	19/75 (25.33)	47/205 (22.93)	
19	50/75 (66.67)	150/205 (73.17)	
20	3/75 (4.00)	4/205 (0.49)	
21	0/75 (0.00)	1/205 (1.95)	

*P*-values < 0.05 were considered statistically significant and appear underlined

Patient characteristics were subdivided into demographic and intraoperative characteristics. Patients were subdivided as suffering NIRS-diagnosed ischemia based on the primary outcome parameter. Continuous variables are presented as mean ± standard deviation; categorical variables are presented as numbers (%). Chi-square, Fisher’s exact or independent samples *t*-tests were used to determine statistically significant differences between de ischemia and non-ischemia groups, depending on the nature of the data. *P* < 0.05 were considered statistically significant

*BSA* body surface area, *NYHA-score* New York heart association score, *Fr* French

**Table 2:** Clinical outcome parameters

	Ischemia (*n* = 75)	No Ischemia (*n* = 205)	*p*-value
Postoperative characteristics			
Acute kidney injury	7/75 (9.33)	13/205 (6.34)	0.374
KDIGO1	4/75 (5.33)	10/205 (4.88)	
KDIGO2	0/75 (0.00)	1/205 (0.49)	
KDIGO3 (without dialysis)	2/75 (2.67)	0/205 (0.00)	
KDIGO3 (with dialysis)	2/75 (2.66)	1/205 (0.49)	
Creatinine levels (mg/dL) postoperative day 1	1.16 ± 0.92	1.05 ± 0.54	0.245
Creatinine levels (mg/dL) at discharge	1.18 ± 1.04	1.08 ± 0.63	0.302
Highest lactate on CPB (mg/dL)	1.68 ± 0.70	1.62 ± 0.60	0.482
First lactate after decannulation (mg/dL)	1.59 ± 0.60	1.50 ± 0.55	0.230
Need for additional peripheral vascular surgery*	0/63 (0)	3/173 (1.73)	0.567

Patients were subdivided as suffering NIRS-diagnosed ischemia based on the primary outcome parameter, defined as a ≥ 15% difference in regional Oxygen Saturation (rSO2) lasting ≥ four consecutive minutes between the cannulated and non-cannulated limb. Continuous variables are presented as mean ± standard deviation, categorical variables are presented as numbers (%), based on the nature of the data. Chi-square or Fisher’s exact, or independent samples *t*-test was used to determine statistically significant differences between de ischemia and non-ischemia groups, depending on the nature of the data

*KDIGO* kidney disease: improving global outcomes

*In total, 44 patients were lost during the follow-up period

**Table 3:** Clinical outcome parameters

	Ischemia (*n* = 18)	No ischemia (*n* = 262)	*p*-value
Postoperative characteristics			
Acute kidney injury	2/18 (11.11)	18/262 (6.87)	0.374
KDIGO1	1/18 (5.56)	14/262 (5.34)	
KDIGO2	0/18 (0.00)	1/262 (0.38)	
KDIGO3 (without dialysis)	0/18 (0.00)	2/262 (0.76)	
KDIGO3 (with dialysis)	0/18 (5.56)	1/262 (0.38)	
Creatinine levels (mg/dL) postoperative day 1	1.63 ± 1.74	1.04 ± 0.50	0.174
Highest lactate on CPB (mg/dL)	1.40 (1.28)	1.50 (0.70)	0.530
First lactate after decannulation (mg/dL)	1.60 (0.75)	1.40 (0.50)	0.226
Need for additional peripheral vascular surgery*	0/16 (0.00)	3/220 (1.36)	1.000

Patients were subdivided as suffering NIRS-diagnosed ischemia based on the secondary outcome parameter, defined as a Tissue Oxygenation Index (TOI) < 50% in the cannulated limb. Continuous variables are presented as means ± standard deviation or median (IQR), categorical variables are presented as numbers (%), based on the nature of the data. Chi-square or Fisher’s exact test, or Mann–Whitney *U* test was used to determine statistically significant differences between de ischemia and non-ischemia groups, depending on the nature of the data

*KDIGO* kidney disease: improving global outcomes

*In total, 44 patients were lost during the follow-up period

**Table 4:** Univariate and multivariate binary logistic regression analysis to identify independent risk factors for the development of distal limb ischemia during MICS

	Univariate			Multivariate		
	OR	*p*-value	95% CI	OR	*p*-value	95% CI
Age	0.968	**0.014**	0.944–0.994	0.960	**0.003**	0.934–0.987
Gender (male)	0.636	**0.134**	0.352–1.149	1.264	0.520	0.619–2.579
BSA (m^2^)	0.331	**0.097**	0.090–1.223	0.255	0.092	0.052–1.251
Peripheral artery disease	1.475	0.417	0.577–3.768			
Surgical type						
V-C	0.691	**0.186**	0.400–1.195	0.700	0.219	0.397–1.236
V-(V + C)	2.174	0.246	0.585–8.069	1.787	0.393	0.472–6.772
C-(V + C)	1.503	0.539	0.410–5.511	1.251	0.740	0.334–4.680
Arterial cannula size (Fr)	1.287	0.401	0.713–2.323			
Duration of cannulation (min)	1.002	0.567	0.996–1.008			

In univariate regression analysis, a *p* < 0.15 is considered statistically significant, and are shown in bold. Statistically significant variables in univariate analysis are included in multivariate regression analyses. *P*-values < 0.05, in multivariate binary regression analysis are considered statistically significant, and are shown in bold. The arterial cannula size was categorized into two groups (small and large) due to too few observations of separate categories

*BSA* body surface area, *V–C* valve surgery versus coronary Artery bypass graft surgery, *V–(V* + *C)* valve surgery versus a combination of valve and coronary artery bypass graft surgery, *C–(V* + *C)* coronary artery bypass graft surgery versus a combination of valve and coronary artery bypass graft surgery, *Fr* French

## Discussion

This prospective observational cohort study focused on distal limb ischemia by continuous, non-invasive tissue oximetry in patients undergoing femoral artery cannulation for MICS. To our knowledge, this is the first large prospective cohort including long-term vascular follow-up in this population. In addition, this is the first study to examine the association between intraoperative NIRS-diagnosed distal limb ischemia and AKI. The incidence of intraoperative NIRS-diagnosed distal limb ischemia varied from 6.4% to 26.79% depending on diagnostic criteria. Multivariate binary logistic regression showed that increasing age is inversely related to the risk of developing distal limb ischemia in the cannulated limb. No association between intraoperative NIRS-diagnosed ischemia and postoperative AKI was found. At completion of the six-month follow-up, we did not find an association between the need for peripheral vascular surgery and intraoperative NIRS-diagnosed distal limb ischemia, neither according to the unilateral ischemia criteria (TOI) nor the bilateral ischemia criteria (ΔrSO2).

Current literature regarding a clinically relevant NIRS cut off value to define ischemia remains indecisive as various authors use different cut off points [23, 24]. Our primary definition of ischemia was based on a study in VA-ECMO patients with femoral artery cannulation. Patton-Rivera found that the positive predictive value for clinical signs of ischemia was 100% in patients suffering rSO_2_-differentials of ≥ 15% lasting ≥ four consecutive minutes disadvantaging the cannulated limb [16]. Our results on incidence were similar to other studies, despite their sample sizes being smaller. Toya et al. retrospectively reviewed 54 patients undergoing MICS with femoral artery cannulation, whereof 17 patients (31.49%) experienced a > 40% rSO_2_-drop compared to baseline, and 11 patients (20.37%) experienced a > 40% rSO_2_-drop compared to the non-cannulated limb [13]. Also, a recent retrospective survey by Shikata et al. showed 21/121 (17.35%) suffered NIRS rSO_2_-differentials > 40% during MICS [11]. Tarui et al. analyzed creatinine phosphorus kinase (CPK) levels in 162 patients undergoing minimally invasive mitral valve repair and found elevated CPK levels in 4.32% of patients [12]. Surprisingly, the authors found no correlation between CPK-levels and musculus gastrocnemius NIRS values measured with NIRO 300 (Hamamatsu^®^, Photonics, Hamamatsu City, Japan). Vida et al. performed an observational study in 50 patients undergoing minimally invasive repair of congenital heart defects. All patients underwent femoral artery cannulation and no association was found between CPK and myoglobin levels and NIRS values (INVOS^®^ 5100 cerebral oximeter; Somanetics Corp, Troy, Michigan). On the other hand, lower limb NIRS values were significantly decreased during CPB in Vida’s study, suggesting a decline in NIRS values to be an earlier sign of ischemia than elevated CPK [25]. Unfortunately, none of cited studies performed a follow-up beyond hospital discharge.

A remarkable novel finding in our study was that baseline NIRS values of patients suffering distal limb ischemia were significantly lower than those in the cohort not suffering distal limb ischemia. Given our study design, a causal relation cannot be determined. Possibilities are lower baseline values to be a risk factor and thus option for preoperative vascular optimization of the patient. However, in a study on NIRS (InSpectra tissue spectrometer^®^, Hutchinson Technology Inc, Hutchinson, Minn) in ambulatory symptomatic peripheral artery disease patients, no difference in baseline was noted between the intermittent claudication group and control group [26].

Investigated risk factors were chosen based on current literature. The study was adequately powered to validate the analysis of all seven risk factors. Multivariate binary logistic regression analysis showed the odds of developing NIRS-diagnosed distal limb ischemia to decrease with increasing age. In their retrospective analysis of 54 patients undergoing MICS with femoral artery cannulation, Toya et al. found no correlation between rSO_2_-differentials and age, gender or duration of surgery. However, mean age in their cohort was 53.2 years with a narrow standard deviation of 1.6 years [13]. This stands in contrast to 68.74 (± 10.3) years in our cohort. Due to the nature of our study, we were unable to explain the protective value of increased age. However, common femoral artery size is known to increase with aging in adults, which might be an important phenomenon for this observation [27]. Another possible reason is younger patients are known to be more prone to reactive vasospasm, which could also lead to decreased NIRS values [28].

The incidence of postoperative AKI in our cohort was 7.14% but was not associated with NIRS diagnosed ischemia. Available data regarding AKI in MICS vary according to the type of surgery, comorbidities and methodology to diagnose AKI [29–31]. We opted for the KDIGO-criteria based on serum creatinine as the risk of adverse outcomes based on urine output after cardiac surgery has been shown to be low [32, 33].

Our study was the first prospective cohort study to perform a follow-up beyond hospital discharge. This is of special interest since the majority of complications after femoral artery cannulation in MICS is noticed only after hospital discharge [9, 14]. At completion of the 6 months follow-up period, 84.28% of patients could be contacted by phone. Of these, three underwent peripheral vascular surgery, whereof one required arterial vascular surgery in the cannulated limb. This patient did not have an intraoperative NIRS decline and did not meet the primary or secondary outcome criteria for ischemia. Furthermore, compared to the prevalence of peripheral artery disease in the general German population (3.14% per year) and the ARIC (Atherosclerosis Risk In Communities)-cohort (9.0% per year), 2 out of 236 cardiac surgical patients requiring peripheral arterial surgery during a six month period might not be surprising [34, 35].

### Limitations

Our study was conducted with a NIRO-200NX device (Hamamatsu^®^, Phototonics K.K., Japan). Different devices emit different wavelengths and use different algorithms for calculation of NIRS values [36]. However, Hyttel-Sorensen showed forearm rSO_2_-values did not significantly differ between NIRO-200NX and INVOS 5100 (Medtronic Somanetics^®^, Troy, Michigan, USA) [37].

Secondly, acute hemodynamic disturbances were not taken into account. Changes in temperature and blood transfusion might alter lower limb rSO_2_ in VA-ECMO patients [38]. On the other hand, Mukaida et al. demonstrated that distal limb rSO_2_ was not affected by hemodilution in patients undergoing cardiac surgery with CPB as measured with a NIRO-200NX device on the tibialis anterior muscle [39]. The same accounts for mean arterial pressure (MAP). However, decreased NIRS values are both described with lower as well as with higher MAP values [40, 41]. In our institution, MAP on CPB is typically targeted at 65 mmHg.

Another limitation is intrinsic to the monocenter design with cannulation and surgical times to be rather short. By consequence, our excellent results might not be generalizable to other cardiac centers.

Lastly, we did not screen for more subjective long-term outcome parameters such as pain on ambulation or paresthesias.

## Conclusion

In conclusion, we found incidence of NIRS diagnosed limb ischemia to vary from 6.42% to 26.79% depending on cut off values. This is line with available literature. Our study was adequately powered for risk factor assessment and younger patients appeared to be at increased risk. We did not find a correlation with postoperative AKI neither important clinical vascular complications on the short nor long term. The added value of distal limb NIRS monitoring only for vascular purposes might be restricted.
